# Chilling temperatures and controlled atmospheres alter key volatile compounds implicated in basil aroma and flavor

**DOI:** 10.3389/fpls.2023.1218734

**Published:** 2023-07-03

**Authors:** Arlan James D. Rodeo, Elizabeth J. Mitcham

**Affiliations:** ^1^ Department of Plant Sciences, University of California, Davis, Davis, CA, United States; ^2^ Institute of Crop Science, College of Agriculture and Food Science, University of the Philippines Los Baños, College, Laguna, Philippines

**Keywords:** basil, chilling injury, volatile profile, controlled atmosphere, low temperature storage, aroma, flavor

## Abstract

Use of basil in its fresh form is increasingly popular due to its unique aromatic and sensory properties. However, fresh basil has a short shelf life and high chilling sensitivity resulting in leaf browning and loss of characteristic aroma. Moderate CO_2_ atmospheres have shown potential in alleviating symptoms of chilling injury in basil during short-term storage but its effect on the flavor volatiles is unclear. Moreover, studies on basil volatile profile as impacted by chilling temperatures are limited. We investigated the response of two basil genotypes to low temperatures and atmosphere modification, with emphasis on the volatile organic compounds responsible for basil aroma and flavor. Leaves were stored for 6 days at 5, 10, or 15°C combined with three different CO_2_ atmospheres (0.04%, 5% or 10%). Basil volatile profile was assessed using headspace solid phase microextraction (HS-SPME) coupled with gas chromatography-mass spectrometry (GC-MS). Leaves suffered severe chilling injury and greater loss of aroma volatiles at 5°C compared to 10°C and 15°C. More than 70 volatiles were identified for each genotype, while supervised multivariate analysis revealed 26 and 10 differentially-accumulated volatiles for ‘Genovese’ and ‘Lemon’ basil, respectively, stored at different temperatures. Storage in 5% CO_2_ ameliorated the symptoms of chilling injury for up to 3 days in ‘Genovese’, but not in ‘Lemon’ basil. Both chilling temperatures and controlled atmospheres altered key volatile compounds implicated in basil aroma and flavor, but temperature had a bigger influence on the observed changes in volatile profile.

## 1 Introduction

Basil (*Ocimum* spp.) is one of the most popular aromatic herbs grown in several regions of the world. It is considered an ultra-niche crop, one of exceptionally high-value that can provide a significant source of income to growers while using minimal land area (Matthews et al., 2018). As a culinary herb, basil leaves provide a crisp and aromatic element to a variety of food and beverage preparations. It is also a source of essential oils, aroma compounds, and valuable phytonutrients known for their antioxidant properties (Simon et al., 1999; Filip, 2017). With the expanding market for fresh aromatic herbs, basil leaves have emerged as a significant segment in the global food ingredients market. The demand for fresh basil has increased immensely through the years due to the shift to healthier and better-tasting food preparations (Curutchet et al., 2014). However, fresh basil has a relatively short shelf life and is very sensitive to chilling temperatures (Cantwell and Reid, 1993; Lange and Cameron, 1997). Storage of basil at temperatures below 12°C during postharvest handling and transport results in chilling injury, characterized by brown discoloration of the interveinal areas of the leaf, stem browning and collapse, loss of glossy appearance, wilting of the leaves and loss of characteristic aroma (Lange and Cameron, 1994; Aharoni et al., 2010).

In basil, a range of management approaches at the farm level have been shown to lessen the severity of chilling injury, such as harvesting in the afternoon (Lange and Cameron, 1994; Aharoni et al., 2010), acclimating plants with less severe low temperatures before and after harvest (Lange and Cameron, 1994; Lange and Cameron, 1997), applying plant hormone treatments (Satpute et al., 2019) or applying artificial lighting during production (Dudai et al., 2017; Jensen et al., 2018). These methods can be supplemented by postharvest strategies to maximize the effect. Controlled atmospheres (CA) and modified atmosphere packaging (MAP) are some techniques used to alleviate chilling injury during transit and storage (Kader et al., 1989). Lowered O_2_ and increased CO_2_ concentrations achieved by atmosphere modification retard deterioration of harvested produce due to effects on physiological processes, including respiration, ethylene production, cellular composition, pathological breakdown, and other metabolic changes (Cantwell and Reid, 1993). Most atmosphere modification studies on basil have centered on extending the shelf life and delaying senescence at or above optimum temperatures for storage, i.e. 12°C (Lange and Cameron, 1998; Amodio et al., 2005; Kenigsbuch et al., 2009; Jirapong et al., 2010; Patiño et al., 2018). However, there is a need to explore the potential of CA storage at chilling temperatures, especially since basil is a chilling-sensitive crop and is being transported commercially in refrigerated vans with other chilling-tolerant commodities. Storage at moderate CO_2_ atmospheres showed promise in alleviating chilling injury in ‘Genovese’ and ‘Thai’ basil (Rodeo and Mitcham, 2022). Whether this alleviation impacts basil aroma and flavor remains unknown.

Volatile organic compounds (VOCs) are responsible for the characteristic aroma and flavor of basil (Chang et al., 2007). Basil volatile profile consists mostly of monoterpenes, sesquiterpenes, and phenylpropenes, and a large number of these secondary metabolites are produced and stored in the glandular trichomes on the surface of the leaves (Gang et al., 2001; Iijima et al., 2004a). Different *Ocimum* species and cultivars vary in their essential oil components, which confer distinct taste and aroma. The sweet basil cultivars have a rich spicy pungent aroma due to the presence of compounds such as linalool, estragole (methylchavicol), 1,8-cineole and eugenol (Simon, 1995; Simon et al., 1999). The aroma of estragole can be compared to that of anise. Linalool and 1,8-cineole respectively give the floral and camphoraceous notes while eugenol is reminiscent of cloves (Burdock and Fenaroli, 2010). Lesser known types and some cultivars of commercial importance can contain a wide range of aromas and unique flavors, including lemon, licorice, or cinnamon (Simon, 1995; Simon et al., 1999).

Since basil is most commonly used as a culinary herb, it is vital that sensory attributes, particularly its aroma and flavor, are preserved because these contribute to the overall eating quality and consumer satisfaction. It is, therefore, important to find the best storage conditions that will limit significant alterations in volatile constituents and hence, flavor. Loss of these volatile organic compounds is among the consequences of chilling injury in fresh herbs (Xiao et al., 2015). Aroma and flavor loss could precede visible chilling symptoms manifestation, and assessment of volatile changes can be utilized as a diagnostic marker for imminent stress which can affect the condition of fresh produce (Cozzolino et al., 2016). Yet, information on the changes in volatile compounds of fresh basil subjected to chilling temperatures is limited. Moreover, the ability of controlled atmospheres to modulate the impacts of low temperature storage on basil volatile compounds has not been documented.

In the present work, we analyzed changes in the volatile compounds of two chilling-sensitive basil genotypes, ‘Genovese’ (*Ocimum basilicum*) and ‘Lemon’ (*Ocimum* × *citriodorum*), stored at different temperatures in an attempt to identify volatile markers implicated in the chilling response of the said basil types. We also explored the potential of moderate CO_2_ atmospheres to alleviate chilling injury in both ‘Genovese’ and ‘Lemon’ and the impact of controlled atmospheres and low temperatures on the concentration of key basil flavor volatiles.

## 2 Materials and methods

### 2.1 Plant material

‘Genovese’ and ‘Lemon’ basil seeds were obtained from Botanical Interests^®^ (Broomfield, CO, USA) and sown in a mixture of peat and sand (1:1 ratio by volume). Approximately 2 weeks after germination, seedlings were transplanted into 3-L pots with the same media and grown inside a climate chamber (BioChambers, Winnipeg, MB, Canada) set at 27 ± 0.2°C and 86 ± 5.6% RH with a 16-h photoperiod and canopy irradiance of 200 µmol m^-2^ s^-1^ provided by cool-white fluorescent lamps (Philips, Cambridge, MA, USA). Sixty days after sowing, leaves from the first four nodes starting from the apex were harvested and randomly packed inside a vented plastic clamshell (18.7 × 12.1 × 8.3 cm). Three clamshells per treatment, each containing approximately 15-30 leaves, depending on leaf size, were used for storage experiments.

### 2.2 Storage conditions

Clamshells containing basil leaves were stored at 5, 10 or 15°C. For each temperature, the leaves were exposed to three atmospheres: 10% CO_2_ + 11% O_2_; 5% CO_2_ + 16% O_2_; and 0.04% CO_2_ + 21% O_2_. Gas mixtures were passed through a gas flow board at a continuous flow rate of 100 mL min^-1^, bubbled through water for humidification, then supplied to plastic bags containing three clamshells each. One flow board delivered gas mixtures to three bags, each serving as a replicate. Gas concentration inside each bag was measured periodically using a CO_2_/O_2_ gas analyzer (Bridge Analyzers, Bedford Heights, OH, USA). Three clamshells (one from each bag) were withdrawn from storage for assessment of chilling injury and analysis of volatile changes after 3 and 6 days, and after an additional 2 days of storage at 20°C in air following 6 days of controlled atmosphere exposure.

### 2.3 Assessment of chilling injury

Visual damage due to chilling injury (i.e. browning of leaves) was assessed using the following scale (Wongsheree et al., 2009): (1) no damage, (2) several dark spots, (3) less than 30% of total leaf area brown, (4) 30–50% of leaf area brown, and (5) more than 50% of leaf area brown. Electrolyte leakage was determined according to the method described by Cozzolino et al. (2016) with slight modification. In brief, approximately 0.5 g of square leaf segments (0.5 cm^2^), collected from three random leaf samples in clamshells previously stored at 5, 10, or 15°C, were washed with ultrapure water and placed in 10 mL of an isotonic solution of 0.1 M mannitol. Sample electrical conductivity was measured using a dual channel benchtop meter (Mettler Toledo, Columbus, OH, USA), after incubation for 30 min on a rotary shaker (Hoefer Scientific Instruments, San Francisco, CA, USA). Total conductivity was recorded following a 48-hour freeze/thaw cycle. Electrolyte leakage was recorded as the percentage ratio of initial over total conductivity.

### 2.4 Volatile analysis by gas chromatography-mass spectrometry

Headspace volatile compound analysis was performed using solid phase microextraction (SPME). Approximately 0.75 g of square leaf segments (0.5 cm^2^) from three random basil leaf samples in a clamshell were immediately placed in a 20-mL headspace vial with a magnetic screw cap lined with a PTFE/silicon septum (Agilent Technologies, Santa Clara, CA, USA). SPME was performed using a divinylbenzene/carboxen on a polydimethylsiloxane (DVB/CAR/PDMS) 50/30 µm fiber (Supelco, Bellefonte, PA, USA) for 30 min at 40°C. The SPME fiber was injected into a gas chromatograph (Agilent Technologies 6890N Network GC System) paired with a 5975B inert XL EI/CI mass selective detector (Agilent Technologies, Santa Clara, CA, USA). The injection port was maintained at 250°C and the volatile compounds were separated in an Agilent DB-WAX Ultra Inert (30 m × 0.25 mm × 0.5 µm) capillary column using helium as the carrier gas at 1.2 mL min^-1^ flow rate. The initial oven temperature was set at 40°C and held for 5 min, then increased to 80°C at a rate of 5°C min^-1^ followed by a 200°C-ramp up at a rate of 10°C min^-1^, and finally up to 250°C at 20°C min^-1^, where it was held for 10 min. Total GC run time was 37.5 min with 1 min post-run time at 260°C. The mass selective detector was operated in electron ionization mode (70 eV) with a full scan mass range of 30–300 m/z (threshold: 150). The transfer line, ion source, and quadrupole temperatures were set at 280, 230, and 150°C, respectively.

Data collection and processing were done with Agilent MSD ChemStation. Volatile compounds were identified by matching their mass spectra to the NIST05 mass spectral database and comparison of their retention times with authentic standards, when available. Further identification was done by comparing their linear retention indices (RI) with those found in the literature, determined relative to the retention time of a C_8_-C_20_ n-alkane series run under the same GC conditions. Relative quantification of volatiles was carried out by comparison of their peak areas to that of stable isotope external standards. Hexanol-*d_13_* (alcohols), octanal-*d_16_* (aldehydes, ketones, and esters), and α-methylstyrene-*d_10_* (terpenoids and phenylpropanoids) (C/D/N Isotopes, Pointe-Claire, QC, Canada) were used to represent the range of compounds found in basil. Standards were run externally to avoid potential competition for SPME adsorption sites between internal standards and native headspace compounds, and to limit any interference with the natural equilibrium that might happen during extraction (Franklin et al., 2017).

### 2.5 Statistical analysis

Volatile concentration data was log-transformed and auto-scaled prior to comprehensive downstream analysis using the web-based software MetaboAnalyst 5.0 (Xia et al., 2009). Univariate analysis was performed to determine the statistical significance and fold change of volatile compounds between chilling and non-chilling temperatures. Multivariate analyses such as Principal Component Analysis (PCA) and Orthogonal Projections to Latent Structures-Discriminant Analysis (OPLS-DA) were also employed to visualize patterns and maximize separation of differential metabolites. Leave-one-out cross-validation (LOOCV) and permutation tests (1000 permutations) were performed to assess the quality of the model and the tendency for data overfitting. The Variable Importance in Projection (VIP) was used to determine the relative importance of each volatile in the OPLS-DA model. Differential volatiles were screened based on the combination of three parameters: VIP ≥ 1, fold change ≥ 2 or ≤ 0.5, and *p-*value (≤ 0.05) adjusted for false discovery rate (FDR).

A three-way ANOVA was performed to determine the effect of storage temperature, atmosphere treatments, and storage duration on the volatile compounds. *Post hoc* analysis was carried out by Tukey’s Honest Significant Difference. PCA, hierarchical cluster analysis, and heat maps were performed and built using R 4.2.0 in RStudio (Posit, PBC).

## 3 Results

### 3.1 Chilling injury parameters as affected by temperature

**Figure 1:**
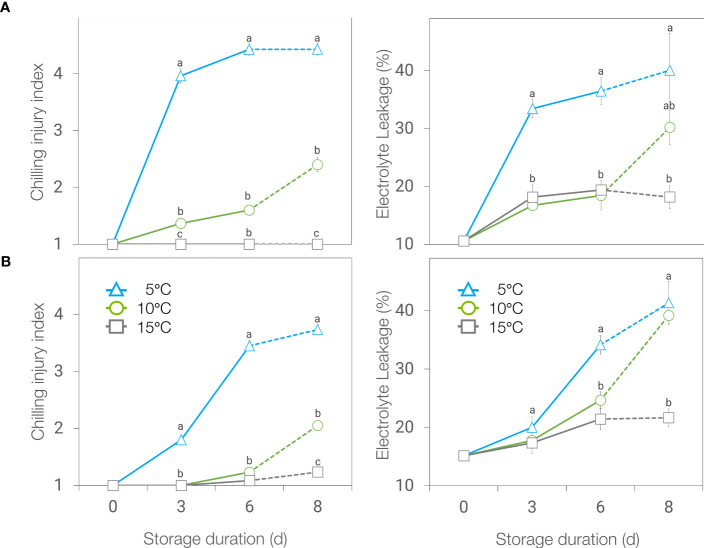
Chilling injury (CI) index (left) and electrolyte leakage (right) of ‘Genovese’ **(A)** and ‘Lemon’ **(B)** basil stored at 5, 10 and 15°C for 6 days + additional 2 days at 20°C (broken lines). Means with the same letter within a storage period are not significantly different (*p* < 0.05).

Chilling injury (CI) development in basil leaves was affected by temperature and the duration of storage, in ‘Genovese’, and by the interaction of both factors in ‘Lemon’ basil. To highlight the sole effect of temperature without atmosphere modification, a separate plot was presented considering only air storage (**Figure 1**). Browning and blackening as a symptom of chilling injury was higher in leaves stored at 5°C compared to the ones held at 10 or 15°C for both basil genotypes throughout storage (**Figures 1A, B**). Electrolyte leakage followed the same trend as CI index, except that after 2 days at 20°C following 6 days storage at chilling temperatures, no difference was found between leaves stored at 5 and 10°C.

### 3.2 Temperature and atmosphere treatment effects on chilling injury parameters

**Figure 2:**
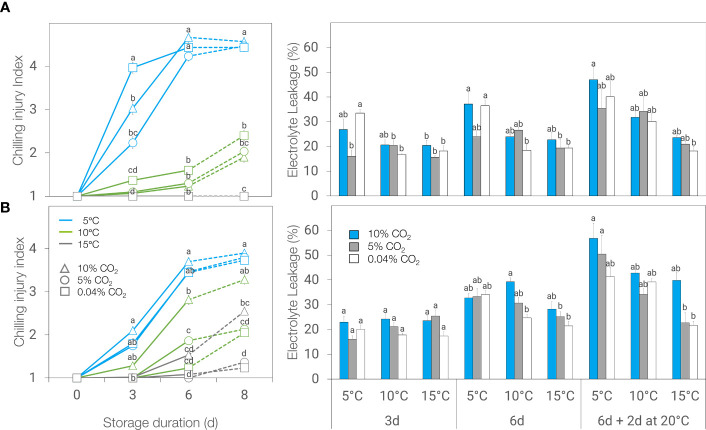
Chilling injury (CI) index (left) and electrolyte leakage (right) of ‘Genovese’ **(A)** and ‘Lemon’ **(B)** basil stored under various CO_2_ atmospheres (0.04, 5 or 10% CO_2_) and temperatures (5, 10, or 15°C) for 3 days, 6 days, and 6 days + additional 2 days at 20°C (broken lines). Means with the same letter within a storage period are not significantly different (*p* < 0.05).

The development of browning symptoms in ‘Genovese’ basil was affected by the atmosphere and the interaction between temperature and duration of storage. Browning symptoms, as a manifestation of CI, were greater at 5°C compared to 10 or 15°C, regardless of atmosphere and duration of storage. There was no significant difference in CI index between basil leaves held at 10 and 15°C, except during post-storage at 20°C for leaves stored under 0.04% CO_2_. Storage in 5% CO_2_ alleviated chilling injury for up to 3 days at 5°C, as shown by lower CI index and electrolyte leakage (**Figure 2A**).

In ‘Lemon’ basil, browning symptom development was affected by the interaction of temperature, atmosphere, and duration of storage. On the other hand, electrolyte leakage was influenced by the duration of storage and the interaction of atmosphere treatment and temperature. Neither 5% nor 10% CO_2_ alleviated chilling injury in leaves stored at 5°C since no differences were observed in browning symptoms or electrolyte leakage among the atmosphere treatments (**Figure 2B**). Storage at 10% CO_2_ even resulted in higher browning development and electrolyte leakage after 6 days of storage at 10°C and after 2 days post-storage at 20°C following storage at 15°C. Unlike ‘Genovese’, leaf browning was observed in ‘Lemon’ basil even at 15°C.

### 3.3 Effect of chilling temperatures on volatile profile

**Figure 3:**
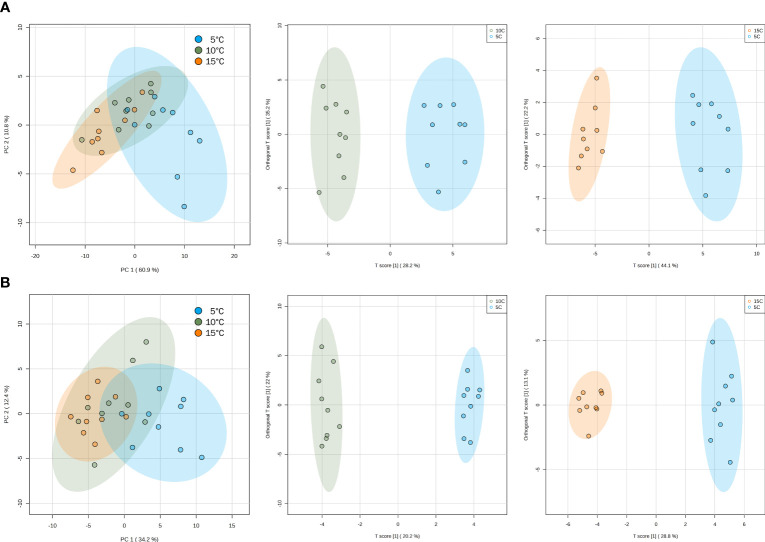
Principal component analysis (PCA) scores plots (left) and orthogonal projections to latent structures discriminant analysis (OPLS-DA) scores plots (middle and right) for ‘Genovese’ **(A)** and ‘Lemon’ **(B)** basil, separated according to the accumulation of volatile compounds at different temperatures. OPLS-DA scores plots are showing 10°C vs 5°C (middle) and 15°C vs 5°C (right) comparison groups, respectively. Confidence regions (95%) are shown in colored ellipses.

More than 70 volatile compounds were identified for each of the basil genotypes under study (**Supplementary Tables 1, 2**). Unsupervised multivariate analysis was done to determine the internal structures of several variables and how these relate based on the principal components. PCA scores plots showed the effect of temperature on the biological replicates (**Figures 3A, B**). For both ‘Genovese’ and ‘Lemon’, there was considerable overlap between samples stored at temperatures of 10 and 15°C, as exemplified by the clustering together of the different replicates. On the other hand, these two groups were held distinct to that of samples stored at 5°C, which tended to move away from the clustering. To further maximize the separation and determine what caused this distinction, orthogonal projections to latent structures-discriminant analysis (OPLS-DA) was done. For both genotypes, a distinct separation in volatile constituents was observed between 10 and 5°C, and between 15 and 5°C, but not between 10 and 15°C (**Figures 3A, B**). Q^2^ values greater than 0.5 in cross-validation and permutation tests suggested an adequately reliable model for both ‘Genovese’ and ‘Lemon’ (data not shown). To screen for differentially accumulated volatiles (DAVs) at different temperatures, the VIP parameter (≥ 1) from the OPLS-DA model was used together with fold change (≥ 2 or ≤ 0.5) and FDR-adjusted *p-*value (≤ 0.05). Based on the thresholds set, there were 28 DAVs between 10 and 5°C and 42 DAVs between 15 and 5°C in the ‘Genovese’ variety, all of which were downregulated when stored at 5°C (**Supplementary Tables 3A, B**). In ‘Lemon’ basil, there were 12 DAVs between 10 and 5°C (9 downregulated and 3 upregulated at 5°C) and 23 DAVs between 15 and 5°C (18 downregulated and 5 upregulated at 5°C) (**Supplementary Tables 4A, B**). There were 26 DAVs found common in both comparison groups for ‘Genovese’, while 10 DAVs were common in both comparison groups for ‘Lemon’. These volatile compounds could play potential roles in the chilling injury response at 5°C and can be starting points for volatile markers to indicate chilling injury in the said genotypes.

### 3.4 VOCs response to combined temperature and controlled atmosphere treatments

The loss of volatile compounds in both genotypes was greater at lower temperatures. A sudden decline in volatile constituents was observed after 3 days at 5°C and continued until day 8 (i.e. withdrawal after 6 days at chilling temperatures and then holding for 2 days at 20°C) (**Supplementary Figure 1**). No significant differences were observed in total combined volatiles among the three CO_2_ atmosphere treatments in ‘Genovese’ (*p* = 0.955) and ‘Lemon’ (*p* = 0.795). More than half of the reduction in volatile compounds was due to decreases in the monoterpenes and sesquiterpenes.

**Figure 4:**
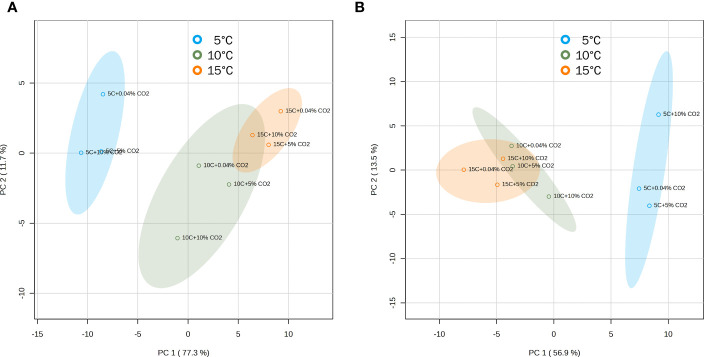
Principal component analysis (PCA) scores plots for ‘Genovese’ **(A)** and ‘Lemon’ **(B)** basil separated according to the accumulation of volatile compounds at different temperature and CO_2_ atmosphere combinations. Leaves are stored under various CO_2_ atmospheres (0.04, 5 or 10% CO_2_) and temperatures (5, 10, or 15°C) for 6 days + additional 2 days at 20°C. Confidence regions (95%) are shown in colored ellipses.

Three-way ANOVA revealed that some VOCs were affected by the interactions between temperature, atmosphere treatment, and duration of storage. However, a lot of variations can be explained by a stronger effect of temperature and duration of storage compared to the controlled atmosphere treatment (**Supplementary Tables 1, 2**). PCA scores plots also supported this observation, separating atmosphere treatments into clusters depending on the accompanying temperature (**Figure 4**). In ‘Genovese’ basil, the first principal component (PC1) explained 77.3% of the variation in the data, clearly separating the 10°C and 15°C temperature groups from the 5°C group. The second principal component (PC2) explained 11.7% of the variation and separated the 10°C group from the 15°C group, as well as the CO_2_ treatment groups at 5°C and 10°C. In ‘Lemon’ basil, PC1 and PC2 explained 56.9% and 13.5% of the variation in the data, respectively, with PC1 separating the 10°C and 15°C temperature groups from the 5°C group, and PC2 separating the atmosphere groups at 5°C. Temperature and CO_2_ treatment groups at 10°C and 15°C tended to cluster together in ‘Lemon’ compared to in ‘Genovese’.

**Figure 5:**
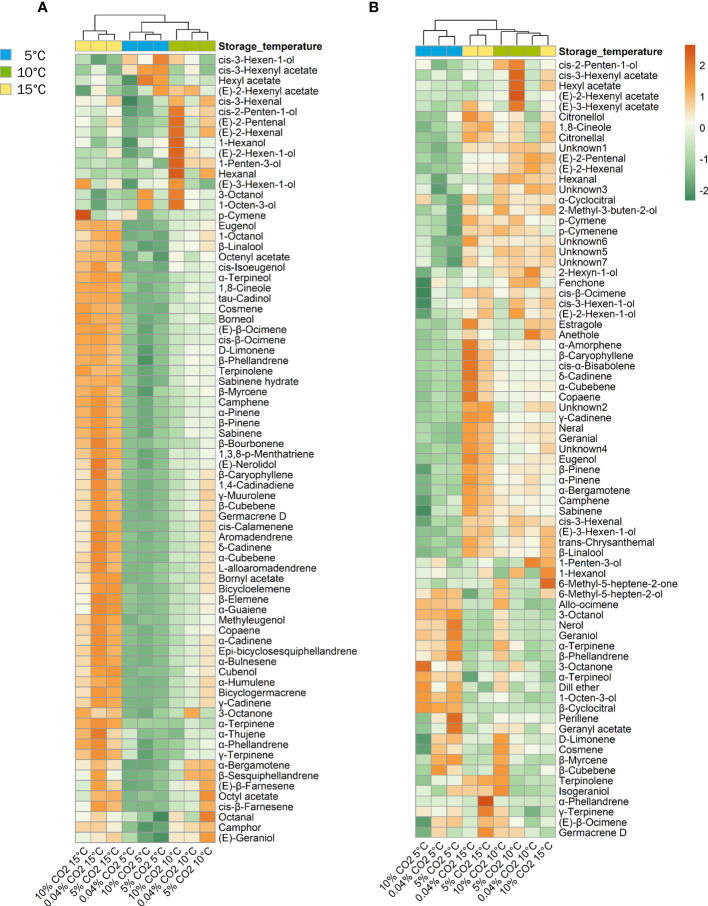
Heatmap of volatile expression for ‘Genovese’ **(A)** and ‘Lemon’ **(B)** basil stored under various CO_2_ atmospheres (0.04, 5 or 10% CO_2_) and temperatures (5, 10, or 15°C) for 6 days + additional 2 days at 20°C. Orange and green colors correspond to upregulation and downregulation, respectively.

Cluster dendogram and heat maps provided insights on the changes in VOC profile expression in the different temperature and atmosphere treatment combinations. Based on the similarities in volatile compound expression in ‘Genovese’ basil, temperature groups were clustered together, as exemplified by the greater loss of terpenoid compounds at 5°C than at 10°C, and higher expression of these volatiles at 15°C (**Figure 5A**). Differences among CO_2_ atmosphere treatments within a temperature group can best be seen at 10°C, with 5% CO_2_ treatment showing higher production of terpenoid compounds and 10% CO_2_ showing high expression of aliphatic alcohols and aldehydes. In ‘Lemon’ basil, cluster separation was observed between the 5°C temperature group and the 10 and 15°C temperature groups, the latter being clustered together, as we observed in the PCA scores plot. Similar to ‘Genovese’, cluster separation was underscored by the largest reductions in most terpenoid compounds and the largest increases of aliphatic and terpene alcohols at 5°C (**Figure 5B**).

### 3.5 Impact of temperature and CO_2_ atmospheres on key flavor volatiles

Out of more than 70 volatile compounds detected and identified for each genotype under study, our analysis was focused mostly on key compounds responsible for basil’s characteristic aroma and flavor. These compounds confer green, woody, fresh, floral and clove aroma and flavor to ‘Genovese’ and a citrusy with a hint of woody, green, spicy, anethole, fresh, floral, and sweet rose aroma to ‘Lemon’ basil (Simon et al., 1999; Al-Kateb and Mottram, 2014; Hammock et al., 2021; Patel et al., 2021).

#### 3.5.1 ‘Genovese’ basil

**Figure 6:**
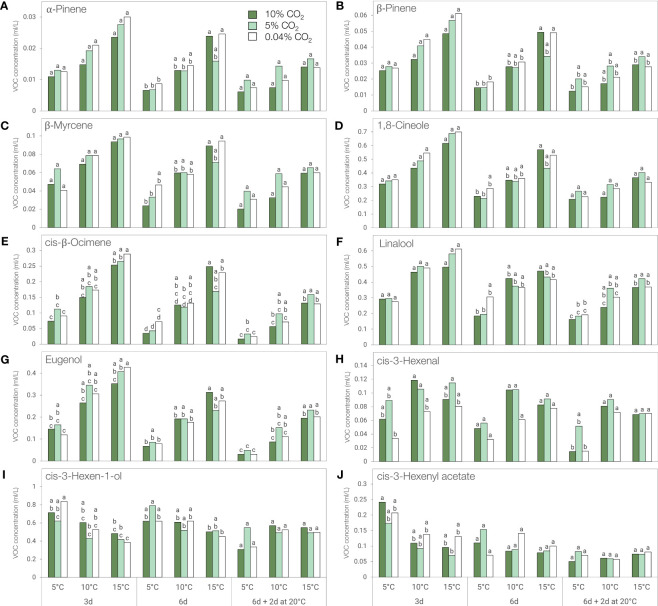
Concentration of key flavor volatiles of ‘Genovese’ basil stored at different temperatures (5, 10, or 15°C) and CO_2_ atmospheres (0.04, 5 or 10% CO2) for 3 days, 6 days, or 6 days + additional 2 days at 20°C. **(A)** α-Pinene, **(B)** β-Pinene, **(C)** β-Myrcene, **(D)** 1,8-Cineole, **(E)** *cis*-β-Ocimene, **(F)** Linalool, **(G)** Eugenol, **(H)** *cis*-3-Hexenal, **(I)** *cis*-3-Hexen-1-ol, **(J)** *cis*-3-Hexenyl acetate. Means with the same letter within a storage period are not significantly different (*p* < 0.05).

The concentration of key flavor volatile compounds α-pinene, β-pinene, β-myrcene, 1,8-cineole, *cis*-β-ocimene, linalool, and eugenol decreased with storage duration and were mostly affected by storage temperature (**Figure 6**; **Supplementary Table 1**). When stored in 0.04% CO_2_, levels of these volatiles were generally lower at 5°C compared to 15°C, although the trend was only statistically significant in α-pinene and β-pinene after 6 days, in *cis*-β-ocimene for all storage periods, and in eugenol after 3 and 8 days (6 days + additional 2 days at 20°C) (**Figures 6A–G**). When stored in 10% CO_2_, the concentrations of α-pinene, β-pinene, β-myrcene, 1,8-cineole, *cis*-β-ocimene, linalool, and eugenol were significantly lower at 5°C compared to 15°C after 6 days (**Figures 6A–G**), while only *cis*-β-ocimene was significantly lower after 3 days (**Figure 6E**). In 5% CO_2_, *cis*-β-ocimene concentration showed a significant decrease when stored at 5°C compared to 15°C after 6 days. Following 2 days of post-storage at 20°C, the levels of *cis*-β-ocimene, linalool, and eugenol were significantly lower at 5°C compared to 15°C whether leaves were held in 5% or 10% CO_2_ (**Figures 6E–G**).

The concentration of *cis*-3-hexenal was affected by the atmosphere, temperature, and the duration of storage. An increase in concentration was observed after 3 days of storage regardless of temperature (**Supplementary Table 1**). Although *cis-*3-hexenal levels were generally higher in 5% and 10% CO_2_, no significant differences were observed between atmospheres for a specific temperature. However, leaves stored at 5°C had significantly lower *cis*-3-hexenal content compared to 15°C in 0.04% CO_2_ and 10% CO_2_ during post-storage at 20°C (**Figure 6H**).

The concentrations of *cis*-3-hexen-1-ol and *cis*-3-hexenyl acetate were affected by the interaction between temperature and duration of storage. A sudden increase was also observed after 3 days but unlike other key volatiles mentioned above, the concentration of these two compounds were generally higher at lower temperatures during the first 6 days of storage (**Figures 6I, J**). Nonetheless, only the levels of *cis*-3-hexen-1-ol in 0.04% CO_2_ showed a statistically significant result after 3 days of storage (**Figure 6I**).

#### 3.5.2 ‘Lemon’ basil

Neral (*cis*-citral) and geranial (*trans*-citral) were the two most abundant volatile compounds in ‘Lemon’ basil, with 10-fold higher concentrations than other volatile compounds. The concentrations of these citral geometric isomers and green leaf volatile *cis*-3-hexen-1-ol were affected by the interaction between the atmosphere, temperature, and duration of storage (**Supplementary Table 2**). Linalool and estragole concentration were affected by temperature and duration of storage, that of β-caryophyllene by the two-way interactions between atmosphere and temperature, and temperature and duration of storage, while *cis*-3-hexenal, and (*E*)-2-hexenal content were influenced by the respective interaction of atmosphere and temperature with duration of storage. Nerol and geraniol concentrations were influenced by the two-way interactions between atmosphere, temperature, and duration of storage.

**Figure 7:**
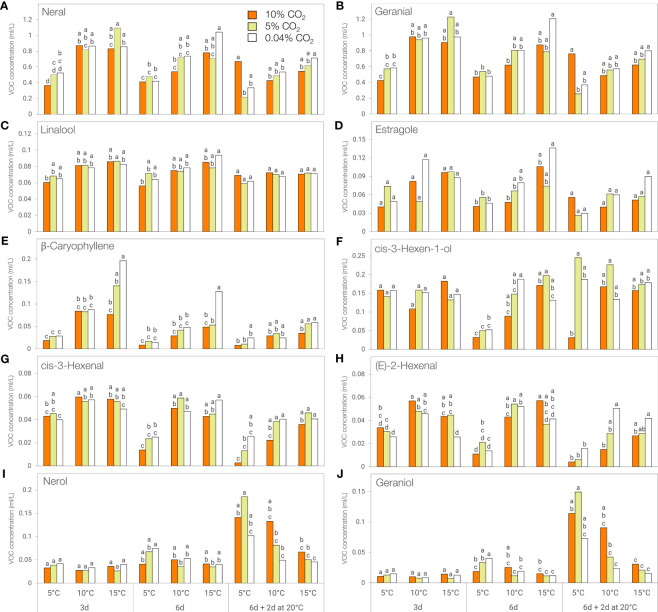
Concentration of key flavor volatiles of ‘Lemon’ basil stored at different temperatures (5, 10, or 15°C) and CO_2_ atmospheres (0.04, 5 or 10% CO_2_) for 3 days, 6 days, or 6 days + additional 2 days at 20°C. **(A)** Neral, **(B)** Geranial, **(C)** Linalool, **(D)** Estragole, **(E)** β-Caryophyllene, **(F)** *cis*-3-Hexen-1-ol, **(G)** *cis*-3-Hexenal, **(H)** **(E)**-2-Hexenal, **(I)** Nerol, **(J)** Geraniol. Means with the same letter within a storage period are not significantly different (*p* < 0.05).

The concentration of these key volatile compounds decreased during storage and was generally lower at 5°C compared to 15°C, except for nerol and geraniol which showed a reverse trend following post-storage at 20°C (**Figure 7**; **Supplementary Table 2**). β-caryophyllene concentration was significantly lower at 5°C compared to 15°C following 3 and 6 days of storage in 0.04% CO_2_ (**Figure 7E**) while a similar trend was observed for neral, geranial, estragole and *cis*-3-hexenal, but only after 6 days of storage in in 0.04% CO_2_ (**Figures 7A, B, D, G**). The concentration of (*E*)-2-hexenal was higher at 10°C compared to 5°C in 0.04% CO_2_ for all storage periods while the same trend was observed in *cis*-3-hexen-1-ol levels but only after 6 days of storage (**Figures 7F, H**).

When stored in 10% CO_2_, significantly lower volatile concentrations were observed at 5°C compared to 15°C in neral and geranial after 3 days, in neral, *cis-*3-hexen-1-ol, and (*E*)-2-hexenal after 6 days, and in *cis*-3-hexenal after 6 and 8 days (6 days + additional 2 days at 20°C) (**Figures 7A, B, F, G, H**). In addition, *cis-*3-hexenal and (*E*)-2-hexenal concentrations were significantly lower at 5°C compared to 10°C after 3 days of storage (**Figures 7G, H**).

When stored in 5% CO_2_, significantly lower volatile concentrations were observed at 5°C compared to 15°C in neral, geranial, and β-caryophyllene after 3 days, in *cis*-3-hexen-1-ol after 6 days, and in *cis*-3-hexenal after 6 and 8 days (6 days + additional 2 days at 20°C) (**Figures 7A, B, E, F, G**). The concentration of (*E*)-2-hexenal was significantly lower at 5°C compared to 10°C after 3 and 6 days of storage (**Figure 7H**). In contrast, nerol and geraniol concentrations were significantly higher at 5°C compared to 10 and 15°C following 2 days post-storage at 20°C (**Figures 7I, J**).

## 4 Discussion

Basil is one of a few chilling-sensitive culinary herbs (Cantwell and Reid, 1993). Chilling injury in basil is characterized by brown discoloration of the interveinal areas of the leaf, stem browning and collapse, loss of glossy appearance, wilting of the leaves and loss of characteristic aroma (Lange and Cameron, 1994; Aharoni et al., 2010). In this study, ‘Genovese’ and ‘Lemon’ basil leaves incurred chilling injury when stored at temperatures below 15°C, with browning symptoms increasing according to the duration of storage. Severe chilling damage, manifested by leaf darkening, was observed at 5°C and this is accompanied by an increase in electrolyte leakage. Progressive loss of membrane integrity and eventual leakage of ions and solutes are among the many physiological disruptions following chilling stress, the manifestations in leaves of which include browning and discoloration. Chilling damage in basil has previously been shown to correlate well with electrolyte leakage (Wongsheree et al., 2009; Cozzolino et al., 2016) and our results also support this finding.

The relative susceptibility of basil to chilling temperatures limits safe transport and storage with other culinary herbs. Since basil is usually transported in packages, atmosphere modification can be easily applied to potentially combat chilling effects. Controlled and modified atmospheres have been found to alleviate chilling injury in a variety of fruits and vegetables (Wang, 1989; Thompson, 2018). In basil, a controlled atmosphere of 1.5% O_2_ + 5% CO_2_ did not prevent the occurrence of chilling injury (Lange and Cameron, 1998). Our previous experiment on ‘Genovese’ and ‘Thai’ basil using 5% and 10% CO_2_ mixed with 16% and 11% O_2_, respectively, showed promise in chilling injury alleviation (Rodeo and Mitcham, 2022). In the current study, 5% and 10% CO_2_ atmospheres alleviated browning symptoms in ‘Genovese’ basil for up to 3 days at 5°C compared to air (0.04% CO_2_) storage. Other than inhibiting ethylene action by acting as a competitive inhibitor (Kader et al., 1989), elevated CO_2_ levels were also found to inhibit the accumulation of reactive oxygen species (ROS), enhance the activities and expression of genes of antioxidant enzymes, and increased accumulation of known antioxidants such as ascorbic acid and glutathione (Liang et al., 2021; Wang et al., 2021). Generation of ROS is one of the key events in chilling injury and its overproduction can lead to progressive loss of membrane integrity due to lipid peroxidation (Sevillano et al., 2009; Heyes, 2018). The aforementioned effects of CO_2_ could have resulted in chilling injury amelioration until such time as the production of ROS exceeded the capacity of the scavenging process, at which time chilling injury symptoms developed.

However, elevated CO_2_ atmospheres were not effective in ameliorating chilling symptoms in ‘Lemon’ basil. Instead, 10% CO_2_ aggravated browning symptoms at 10°C after 6 days and at 15°C following 2 days of post-storage at 20°C. Wongsheree et al. (2009) found that ‘Lemon’ basil was more prone to leaf blackening than holy and sweet basil types. This was also confirmed in our previous experiments comparing five different commercial basil varieties and species, including ‘Lemon’ and ‘Genovese’, that indicated there is a differential chilling sensitivity among basil types (unpublished data). The current study revealed that ‘Lemon’ basil is also more sensitive to high CO_2_ atmospheres. The browning symptoms observed cannot be solely ascribed to the effect of chilling, but also to CO_2_ injury. The combination of the two stresses could be too much for a sensitive genotype such as ‘Lemon’, resulting in intensified symptoms. In ‘Napoletano’ basil, 10% CO_2_ likewise caused serious leaf browning even when stored at an otherwise safe temperature of 12°C (Amodio et al., 2005). Sweet basil stored at 20°C under atmospheres containing more than 10% CO_2_ was found to have a reduced shelf life due to brown spotting and CO_2_ injury (Lange and Cameron, 1998). The relative susceptibility of different basil types to elevated levels of CO_2_ that resulted in injury underscored the need for dynamic CA or MAP systems that will not only cater to changing environmental conditions and a commodity’s metabolic state, but also to species and cultivar differences (Saltveit, 2003).

Aside from leaf browning and discoloration, chilling injury is also accompanied by the loss of volatile organic compounds (Farneti et al., 2015; Wang et al., 2015). Major alterations in the contents of these compounds can result in a significant reduction in flavor quality, a characteristic that is most valued in culinary herbs such as basil (Zhang et al., 2016). Our present work revealed significant changes in the volatile profile of two chilling-sensitive basil genotypes, ‘Genovese’ and ‘Lemon’. Compared to 10 and 15°C, storage at 5°C resulted in lower concentration of a number of volatile compounds deemed important for basil flavor. Differentially expressed volatiles between 5°C and relatively higher chilling temperatures (10°C and 15°C) suggested that fold decreases in storage temperature can lead to greater reductions in volatile constituents. Cozzolino et al. (2016) identified 10 volatile compounds considered to be potential markers of chilling injury in three sweet basil cultivars (‘Italico a foglia larga’, ‘Cammeo’ and ‘Italiano classico’). Our results indicated 26 and 10 differentially accumulated volatiles (DAVs) in ‘Genovese’ and ‘Lemon’, respectively. These compounds were found to be downregulated in ‘Genovese’ and ‘Lemon’ stored at 5°C, with the exception of 6-methyl-5-hepten-2-ol, which was upregulated in ‘Lemon’ basil at 5°C. Since these volatiles were found to be greatly affected by chilling based on the variable importance in projection (VIP) and fold-changes, we can therefore consider them as potential volatile markers for chilling stress in ‘Genovese’ and ‘Lemon’ basil. It is interesting to note that 80% of DAVs in ‘Genovese’ and 50% of DAVs in ‘Lemon’ were sesquiterpenes which could suggest that the synthesis of this group of volatile compounds can be greatly affected by chilling. Sesquiterpenes (C_15_) are derived from isopentenyl diphosphate (IPP) and its isomer dimethylallyl diphosphate (DMAPP), produced by the cytosolic mevalonate (MVA) pathway. On the other hand, monoterpenes (C_10_) are derived from IPP and DMAPP, produced by the parallel methylerythritol (MEP) pathway situated in the plastids (Dudareva et al., 2004; Iijima et al., 2004a). Compartmentalization and synthesis location might have played a role in the relative differences between the response of these two groups of terpenoid compounds.

Studies on the ability of controlled atmospheres to modulate the impacts of chilling temperatures on volatile and flavor compounds of fresh basil are very limited, if not non-existent. Several factors can influence the fate of volatile compounds in the harvested plant; among these are storage methods and postharvest techniques employed (Figueiredo et al., 2008). In basil, controlled atmospheres (3% CO_2_ + 2% O_2_) did not alter volatile composition, but concentrations decreased with duration of storage at 12°C (Amodio et al., 2005). Storage at 4°C compared to 12°C resulted in lower concentrations of volatile compounds in three basil cultivars (Cozzolino et al., 2016). Our results agree with the above studies where volatile abundance generally decreased with time and lower storage temperatures. Storage at 5% CO_2_ was able to mitigate some volatile losses resulting from chilling, especially during the first 3 days of storage, which coincided with visible chilling symptom alleviation in ‘Genovese’ (**Figures 2**, **6**). This was also evident in 10°C storage as shown by the upregulation of volatile abundance with 5% CO_2_ compared to air or 10% CO_2_ (**Figure 5A**).

While volatile changes in fruits post-harvest depend on maturity and ripening processes, this does not apply to basil and other herbs. Instead, storage conditions and leaf morphology could play critical roles in volatile compound dynamics. Most volatile compounds in basil are synthesized and stored in the glandular trichomes and released following environmental cues and mechanical damage (Holopainen and Gershenzon, 2010). Having these external secretory structures makes basil more vulnerable to evaporative volatile losses and transformations, especially during handling and storage (Combrinck et al., 2007; Figueiredo et al., 2008). In this study, multivariate analyses also revealed that temperature was the major driving force for basil volatile changes rather than atmosphere. Storage at 15°C in air resulted in a gradual loss of volatile compounds with time, capped with a further decline following transfer to 20°C. On the other hand, storage at lower temperatures led to a sudden reduction in volatile compounds. This pattern was generally true even for higher CO_2_ atmosphere storage. PCA and cluster analysis showed groupings mostly according to storage temperature. There was a very distinct separation between basil stored at 5°C and those that were held at relatively higher temperatures of 10 and 15°C in the 3 atmospheres (**Figures 4**, **5**). This suggests that for a chilling-sensitive commodity such as basil, most of the volatile losses can be attributed to storage at temperatures significantly below the chilling threshold. Most studies on basil chilling injury denote the critical temperature as 12°C, below which chilling damage will be expressed (Lange and Cameron, 1994; Meir et al., 1997; Wongsheree et al., 2009; Aharoni et al., 2010; Cozzolino et al., 2016), be it visible (browning and wilting) or invisible (loss of volatiles and characteristic aroma). Holding basil closer to this threshold temperature is important for volatile compound preservation. Interestingly, controlled atmosphere storage at 10°C led to an increase in volatile aldehydes and their alcohol derivatives which are mostly products of the oxylipin pathway. In mango peel, chilling stress also increased the production of these volatiles and was suggested to be implicated in abiotic stress signaling (Sivankalyani et al., 2017). This might also be the case in basil. The elevated production of these volatiles at median temperatures could signal impending stress to enable activation of defense responses.

The characteristic aroma and flavor of ‘Genovese’ basil leaves can be attributed to the presence of key volatile compounds. Consistent with the findings of Chang et al. (2007), the major volatile compounds observed in ‘Genovese’ basil consisted of a 3:3:2 ratio of 1,8-cineole, linalool, and eugenol. A pure standard mixture of these volatiles that we made in a similar ratio recreated the major aroma notes of ‘Genovese’ basil, as perceived by a trained panel (unpublished data). Cineole (eucalyptol) has a camphoraceous aroma and a fresh, cooling taste, suggestive of mint. Linalool is responsible for the sweet floral aroma in basil, while eugenol has a strong clove-like odor and a spicy, pungent taste (Burdock and Fenaroli, 2010). Other compounds that have a significant impact on the aroma and flavor of ‘Genovese’ basil are α-pinene and β-pinene (earthy/pine/woody), β-myrcene (spicy/balsamic), and *cis*-β-ocimene (floral/woodsy). Green leaf volatiles such as *cis*-3-hexenal, *cis-*3-hexen-1-ol, and *cis*-3-hexenyl acetate provide basil with green, grassy, and fruity aroma, respectively. Since culinary herbs such as basil do not have a substantial amount of sugars and acids, loss of these volatile compounds leads to a significant amount of flavor loss (Zhang et al., 2016).

Storage at low temperature, especially at 5°C, accelerated the decrease in the levels of 1,8-cineole, linalool, eugenol, β-myrcene, *cis*-β-ocimene, α-pinene and β-pinene in ‘Genovese’. With the exception of eugenol, all the other volatile compounds mentioned are monoterpenes and therefore share the same biosynthetic pathway. Monoterpenes are derived from geranyl diphosphate (GDP), and produced from the condensation of IPP and DMAPP that are mainly formed in the plastid through the MEP pathway. GDP then undergoes several transformations by a large family of enzymes known as terpene synthases giving rise to different monoterpenes (Bohlmann et al., 1998; Dudareva et al., 2004). Eugenol, on the other hand, is derived from the amino acid, phenylalanine, through the phenylpropanoid pathway, and its biosynthesis proceeds *via* the reduction of a coniferyl alcohol ester by the eugenol synthase enzyme (Gang et al., 2001; Koeduka et al., 2006). The loss of these volatile compounds during subsequent low temperature storage is related to reduced expression levels of genes coding for important enzymes in the metabolic pathway. For instance, the expression of a linalool synthase gene coding for the enzyme responsible for the formation of linalool in a single-stage reaction from GDP was downregulated by chilling temperature in papaya fruit, resulting in impaired linalool production (Gomes et al., 2016). In tomato, significant reduction in volatile concentrations at 5°C was correlated with lower transcript abundance of genes coding for enzymes and products essential for volatile biosynthesis, leading to reduced flavor quality (Zhang et al., 2016).

In contrast, the concentrations of *cis*-3-hexen-1-ol and *cis*-3-hexenyl acetate were generally higher after 3 days at 5°C than at 15°C, while storage in 5% CO_2_ generally maintained the levels of *cis-*3-hexenal regardless of temperature. These green leaf volatiles are derived from the oxygenation of C_18_ unsaturated fatty acids through the action of lipoxygenase (LOX), hydroperoxide lyase (HPL), and alcohol dehydrogenase (ADH) enzymes *via* the oxylipin pathway (Schwab et al., 2008; Bai et al., 2011). The increase in these volatiles at low temperatures might be due to an increase in substrate concentration during chilling since C_6_ volatile aldehydes production is likely determined by substrate availability rather than HPL activity abundance (Vancanneyt et al., 2001). Moreover, the levels of C_18_ unsaturated fatty acids increased in basil leaves after 2 days of storage at 4°C (Wongsheree et al., 2009). Eventual conversion of *cis*-3-hexenal to *cis*-3-hexen-1-ol by ADH activity, and a decrease in ester levels could also explain the high levels of volatile alcohol particularly in leaves stored at 0.04% CO_2_ (Schwab et al., 2008; Farcuh and Hopfer, 2023).

Unlike in ‘Genovese’ basil, green leaf volatiles in ‘Lemon’ basil were affected by controlled atmosphere storage, aside from temperature and duration of storage. Higher CO_2_ atmospheres of 10% resulted in increased leaf browning in ‘Lemon’ basil, even at 10°C, and this highly impacted the levels of *cis*-3-hexenal, (*E*)-2-hexenal, and *cis*-3-hexen-1-ol which impart fresh green and grassy aromas to basil leaves. Lower concentrations of C_6_ volatile aldehydes and alcohol after 6 days of storage at 5°C might be due to downregulation of LOX and HPL gene expression, as has been previously observed in other fresh commodities (Bai et al., 2011; Zhang et al., 2011). Moreover, the increase in *cis*-3-hexen-1-ol after 2 days at 20°C following 6 days of chilling exposure was accompanied by a decrease in the concentration of *cis*-3-hexenal, which hinted to a possible recovery of function of ADH, enabling the conversion of said C_6_ aldehyde to its corresponding alcohol.

Linalool concentration in ‘Lemon’ basil was also reduced by lower temperature, and storage in 10% CO_2_, which enhanced leaf browning, also led to a significant loss in linalool content. Similarly, estragole, a volatile with anise-like flavor, was also significantly reduced in basil leaves after 6 days of storage at 5°C. Just like eugenol, estragole is derived from phenylalanine through the phenylpropanoid pathway and is synthesized by an additional transfer of a methyl group to chavicol by a specific *o*-methyltransferase (Lewinsohn et al., 2000; Gang et al., 2002). Aside from activity suppression of enzymes in the phenylpropanoid pathway, it is highly possible that *o-*methyltransferase activity was also downregulated resulting in a decrease in estragole levels since some methyltransferases have been found to be repressed by chilling in other crops, such as wheat (Onyemaobi et al., 2022).

β-Caryophyllene, one of the volatile compounds associated with the flavor of black pepper, gives ‘Lemon’ basil a spicy and woody note (Burdock and Fenaroli, 2010). The concentration of this volatile declined significantly following chilling exposure early in storage, when visible chilling symptoms were still limited. Thus, β-caryophyllene can be considered as an early diagnostic marker of chilling injury in ‘Lemon’ basil. As a sesquiterpene, this volatile is derived from farnesyl diphosphate (FDP), another condensation product of IPP and DMAPP, which are mainly produced in the cytosol *via* the mevalonate pathway. A caryophyllene synthase from the large terpene synthases family then generates caryophyllene from FDP, and is likely downregulated during chilling exposure (Bohlmann et al., 1998; Dudareva et al., 2004).

Chilling temperatures also led to a significant reduction in citral concentration in ‘Lemon’ basil. Citral, which exists in two geometric isomers, neral and geranial, is the most abundant volatile compound and is mainly responsible for the lemon-like aroma and flavor of ‘Lemon’ basil. In ‘Sweet Dani’, another basil cultivar known for its lemony flavor, more than 99% of the monoterpenes found are comprised of neral and geranial, along with the monoterpene alcohols nerol and geraniol (Iijima et al., 2004b). Nerol and geraniol have a characteristic sweet, rose-like odor and bitter flavor (Burdock and Fenaroli, 2010). Geranial is produced from the oxidation of geraniol by alcohol dehydrogenases, which then undergo non-enzymatic conversion *via* keto-enol tautomerization to neral. The reduction of neral by dehydrogenase enzymes will in turn produce nerol (Iijima et al., 2004b; Iijima et al., 2006). Storage of ‘Lemon’ basil leaves at 5°C could have resulted in reduced activity of geraniol synthase and alcohol dehydrogenases, resulting in lower concentrations of citral. It is noteworthy that the concentrations of nerol and geraniol increased after 2 days post-storage at 20°C following 6 days at 5°C, especially after storage in elevated CO_2_ atmospheres. Chilling injury may have triggered accumulation of said monoterpene alcohols through increased concentrations of reduced cofactors, such as NADPH, facilitating the reduction of neral and geranial to their corresponding alcohols (Iijima et al., 2006).

## 5 Conclusion

Chilling injury in basil can be manifested both by visible (browning and leaf discoloration) and invisible (loss of volatile compounds responsible for aroma and flavor) damage. Chilling damage is more severe when leaves are stored at temperatures far lower than the threshold temperature. Storage at 5°C resulted in more chilling injury than at 10°C or 15°C, but injury occurred in all three temperatures. Storage at 5°C also resulted in differential accumulation of 26 and 10 volatiles in ‘Genovese’ and ‘Lemon’ basil leaves, respectively. Out of these differentially expressed volatiles, *cis*-3-hexenal, eugenol, and germacrene D have significant potential as diagnostic markers for chilling stress in ‘Genovese’ based on their VIP values and relative importance to flavor perception. For ‘Lemon’ basil, *cis*-α-bisabolene, β-caryophyllene, and estragole have similar marker potential. Controlled atmosphere storage with 5% CO_2_ alleviated chilling injury by reducing visible symptoms and maintaining volatile concentrations in ‘Genovese’ basil for up to 3 days, but did not have a similar effect in ‘Lemon’ basil. Volatile changes were more influenced by storage temperature than the accompanying atmosphere. Our study demonstrated that the impact of low temperatures on volatile abundance can be modulated by moderate CO_2_ atmospheres (i.e., 5%), albeit for a short period. This modulation was also found to be cultivar- and species-dependent, as susceptibility to CO_2_ injury limits its potential application. Sensory evaluation based on descriptive analysis can be done to further evaluate this effect on basil flavor quality in relation to actual human perception.

## Data availability statement

The original contributions presented in the study are included in the article/**Supplementary Material**. Further inquiries can be directed to the corresponding author.

## Author contributions

AR and EM conceptualized the research and designed the experiments. AR carried out the experiments, analyzed the data, and wrote the original draft. EM reviewed and revised the manuscript. Both authors contributed to manuscript revision and approved the submitted version.
